# Medicinal Plants: A Source of Anti-Parasitic Secondary Metabolites

**DOI:** 10.3390/molecules171112771

**Published:** 2012-10-31

**Authors:** Michael Wink

**Affiliations:** Institute of Pharmacy and Molecular Biotechnology, INF 364, Heidelberg University, D-69120 Heidelberg, Germany; Email: wink@uni-hd.de; Tel.: +49-6221-544881; Fax: +49-06221-544884

**Keywords:** protozoa, parasites, medicinal plants, secondary metabolites, molecular targets

## Abstract

This review summarizes human infections caused by endoparasites, including protozoa, nematodes, trematodes, and cestodes, which affect more than 30% of the human population, and medicinal plants of potential use in their treatment. Because vaccinations do not work in most instances and the parasites have sometimes become resistant to the available synthetic therapeutics, it is important to search for alternative sources of anti-parasitic drugs. Plants produce a high diversity of secondary metabolites with interesting biological activities, such as cytotoxic, anti-parasitic and anti-microbial properties. These drugs often interfere with central targets in parasites, such as DNA (intercalation, alkylation), membrane integrity, microtubules and neuronal signal transduction. Plant extracts and isolated secondary metabolites which can inhibit protozoan parasites, such as *Plasmodium*, *Trypanosoma*, *Leishmania*, *Trichomonas* and intestinal worms are discussed. The identified plants and compounds offer a chance to develop new drugs against parasitic diseases. Most of them need to be tested in more detail, especially in animal models and if successful, in clinical trials.

## 1. Introduction

During the evolution of humans a broad set of parasites have evolved, that use us as a host organism. Usually a parasite will not kill its host (at least not immediately), as this would by an evolutionary dead end for a parasite. However, most parasites are either unpleasant for us (think of lice and fleas) or weaken our health (most internal parasites). However, some parasitic infections, such as malaria, trypanosomiasis or Chagas can be deadly if the patients are not treated with adequate therapeutics. Because humans usually live in close proximity and often without good hygienic conditions the transmission of parasites within a human population is often facilitated.

It is very likely that humans have always tried to get rid or minimize the impact of parasites. External parasites (ectoparasites) could be reduced or eliminated mechanically. This could be done individually or in groups. Grooming is a common behaviour in primates and monkeys delouse each other in consequence. Humans probably did the same. More complicated to treat were internal parasites (endoparasites). We know that humans have used medicinal plants for several thousands of years to treat illness and health disorders [1]. It is likely that humans also identified plants which were useful as antiparasitic drugs. Even today, infections by parasites are often treated by plant products or secondary metabolites isolated from them. Circumstantial evidence suggests that chimpanzees, our closest primate relatives, selectively eat medicinal plants when suffering from infections.

Unfortunately, infections by endoparasites can hardly be prevented by vaccination. Even for malaria, one of the most common parasitic diseases which infects over 200–300 million people and kills more than 1 million per year, an effective vaccine is not (yet) available because the parasites have clever strategies to outcompete our immune system, for example by continuously changing their surface coatings.

Medicinal chemists have synthesized a number of drugs which can be used against many but by far not all endoparasites. A major problem is that many of these drugs were developed many years ago and some parasitic strains have become resistant to them. The development of new antiparasitic drugs has not been much of a priority for the pharmaceutical industry because many of the parasitic diseases occur in poor countries where the populations cannot afford to pay a high price for the drugs. Thus an investment in drug development against parasitic diseases is a risky affair.

An alternative to synthetic drugs is the search for anti-parasitic plant extracts or secondary metabolites derived from them. Natural products still play an important role in therapy: between 1981 and 2006, 1,184 new drugs were registered of which 28% were natural products or their derivatives. Another 24% of the new drugs had pharmacophores (*i.e.*, functional groups with pharmacological activity) derived from natural products [2]. A good starting point to find antiparasitic natural products would be traditional medicinal plants, such as those known from Asia, Africa or America [1] that have been employed to treat infections. 

Many promising results have been obtained so far to kill the parasites or their vectors *in vitro*, however a translation of these results into clinical practice is a neglected field. In this review, special focus will be on medicinal plants used in traditional medicine against parasitic infections. It is out of scope of this review to mention all the natural products individually that have been tested and which exhibit some sort of antiparasitic activity. If available, references to more specific reviews are provided. Furthermore, tropical diseases caused by viruses, bacteria and fungi will not be addressed in this review although plant-derived drugs can provide interesting candidates for therapy [3].

## 2. Human Parasites and Parasitic Diseases

Many parasitic infections are the cause of tropical diseases, such as malaria, trypanosomiasis, leishmaniasis, Chagas disease, schistosomiasis, onchocerciasis, lymphatic filariasis, and helminthiases. Parasites are responsible for probably more than 1–2 billion infections, which lead to several million deaths every year [4]. In addition to these economically important parasitic diseases, a number of ectoparasites affect human health, which include mites (*Sarcoptes scabiei* causing scabies), lice (*Pediculus capitis*; *Phthirus pubis*), bed bugs (*Cimex lectularius*, *C. hemipterus*), fleas (*Pulex irritans*, *Tunga penetrans*), and several myiasis-producing diptera (*Chrysomya*, *Cochliomya*, *Wohlfahrtia*, *Sarcophaga*, *Dermatobia*, *Cuterebra*, *Gasterophilus*, *Hypoderma*, *Oestrus*). An overview on important endoparasites, their vectors, distribution, and disease symptoms is given in Table 1 [4,5] in order to understand the challenge which we are faced worldwide. Many human parasites are transmitted by arthropod vectors, which could also be a target for secondary metabolites with insecticidal properties [6,7].

**Table 1:** Examples (a selection) of important human endoparasites [4].

Parasite	Disease (estimated number of infections)	Vector (hosts); route of transmission	Distribution	Symptoms
**Protozoa**				
**Apicomplexa**				
*Babesia* spp.	Piroplasmosis (rare)	Ticks (*Ixodes*); bites	North America	Anaemia, damage of immune system
*Plasmodium* **	Malaria (>250 million)	Mosquitos**	Tropics and subtropics	Anaemia, enlarged liver and spleen, high fever, jaundice, haemorrhage, haemoglobinuria (“blackwater fever”); blockage of cerebral capillaries (*P. falciparum*)
(*P. vivax* , *P. ovale* ,				
*P. malariae* ,				
*P. knowlesi* ,		( *Anopheles* , *Nyssorhynchus* ,		
*P. falciparum* )		*Cellia* , *Kerteszia* ); bites		
*Toxoplasma gondii*	Toxoplasmosis	Main host are cats; infection of humans from faeces	Worldwide	Flu-like symptoms; cysts in muscle and neural tissues; encephalitis, serious danger for developing foetus (abortion, malformations)
**Trypanosomatida**				
*Trypanosoma brucei*;**	African trypanosomiasis (>500,000)	Tsetse flies (*Glossina*); bites	Tropical Africa	Fever, rash, lymphoadenopathy, sleeping sickness (waste, comatose)
*T. b. gambiense* **				
*T. b. rhodesiense*				
*T. b. brucei*	Nagana (only cattle)	Tsetse flies (*Glossina*); bites	Tropical Africa	Loss of cattle; symptoms as in humans
*Trypanosoma cruzi*	Chagas disease (10 million)	Bugs of the family Reduviidae (*Rhodnius*, *Triatoma*, *Panstrongylus*); bites	Central and South America	Local tissue lesions of eyes (Romana’s sign), myocarditis, cardiomegaly, megaoesophagus, megacolon
*Leishmania donovani*	Visceral leishmaniasis (kala-azar) (15 million)	Flies (*Phlebotomus*,	N-Africa, Eurasia	Enlargement of liver and spleen, fever, dermal lesions, dermal nodules
		*Lutzomyia* ); bites	S America	
*Leishmania tropica*	Cutaneous leishmaniasis (Old world)	*Phlebotomus*; bites	Eurasia, Africa	Ulcerative lesions, mucocutaneous lesions
*L. major*				
*L. infantum*				
*Leishmania mexicana* and several others	Cutaneous leishmaniasis (New world)	*Lutzomyia*; bites	Central and Southern America	Ulcerative lesions, mucocutaneous lesions
**Amoebida**				
*Entamoeba histolytica* and other species	Amoebiasis (70,000 deaths/year)	Infection from contaminated water or food	Worldwide	Dysentery, destruction of intestinal tissues, fever, liver and lung abscess
**Diplomonadida**				
*Giardia lamblia*	Giardiasis (2 million infections/year in USA)	Infection from contaminated water	Worldwide	Infection of duodenal and jejunal mucosa; diarrhoea, fever
**Trichomonadida**				
*Trichomonas vaginalis* **	Trichomoniasis (180 million each year)	Sexual transmission	Worldwide	Mucosal tissue of genital tract
*Trichomonas hominis*				
**METAZOA**				
**Nematoda**				
**Filarioidea**				
*Wucheria bancrofti Brugia spp.* **	Lymphatic filariases, elephantiasis (120 million)	Mosquitos (*Aedes*, *Culex*, *Mansonia*); bites	Tropical Africa, Asia, America	Infection of lymphatic system; enlargement of lymph nodes
*Mansonella spp.*				
*Loa loa*	Loaiasis (33 million)	*Chrysops*	Central Africa	Female worms migrate through tissues and the eye
*Onchocerca volvulus*	Skin filariases; onchocerciasis; river blindness (>17 million)	Flies (*Simulium* spp.); bites	Mostly tropical Africa and America	Formation of large nodules under skin or in eyes (causing blindness)
**Trichuroidea**				
*Trichinella spiralis*	Trichinosis (50 million)	Eating of infected muscles e.g., from pigs; bites	Worldwide	Invades muscular tissue, fever, myalgia, malaise and oedema
*Trichurus trichiura*	(500 million)	Infection from contaminated soil	Worldwide	Intestinal infection
**Rhabditoidea**				
*Strongyloides stercoralis*	Strongyloidiasis (70 million)	Infection from contaminated soil	Subtropics, tropics worldwide	Intestinal infection, anaemia; migrating larvae in skin
**Ancylostomatoidea**				
*Ancylostoma duodenale*;**	Hookworm infection (700–900 million)	Infection from contaminated soil	Subtropics, tropics worldwide	Intestinal infection, anaemia, migrating larvae in skin
*Necator americanus*				
**Oxyuroidea**				
*Enterobius vermicularis*	Thread or pinworm (400 million)	Infection from contaminated humans	Worldwide	Intestinal infection
**Ascaridoidea**				
*Ascaris lumbricoides*	Ascariasis (800–1000 million)	Infection from contaminated soil	Worldwide	Intestinal infection, migrating larvae in various tissues
**Dracunculoidea**				
*Dracunculus medinensis*	Dracunculiasis; guinea worm infection (<3 million)	Copepods as intermediate host	Africa, Asia	Infects skin; female worms can reach a length of 100 cm
**Plathelmintes** **Trematoda**				
**Schistosomatoidea**				
*Schistosoma mansoni* **	Schistosomiasis (200 million)	Water snails as intermediate host	Tropical and subtropical Africa, S America and E Asia	Dermatitis, infects liver, granuloma formation in liver, liver fibrosis, enlarged spleen
*S. japonicum*				
*S. haematobium*	Schistosomiasis (80 million)	Water snails as intermediate host	Africa	Infection of bladder, haematuria
**Echinostomatoidea**				
*Fasciola hepatica*	Fasciolopsiasis (2.4 million)	Water snail (*Lymnaea*) as host	Worldwide	Infection of liver
*F. gigantica*				
**Opisthorchioidea**				
*Opisthorchis felineus*	Opisthorchiasis, liver fluke (10 million)	Water snails as intermediate host; infection from infected fish	E Europe, Central and Eastern Asia	Infection of liver and gall bladder
*O. viverrini*				
*O. sinensis* (*syn. Clonorchis*)	Clonorchiasis (35 million)	Water snails as intermediate host; infection from infected fish	China, Japan	Infection of liver, fibrosis, carcinoma
**Plagiorchioidea**				
*Paragoniumus westermani*	Paragonimiasis (20 million)	Water snails and craps as intermediate hosts; infection from infected meat	Tropics of Africa, America and E Asia	Infection of internal organs, including lungs and brain
*P. mexicanus* and other species				
**Platyhelminthes/Cestoda**				
*Diphyllobothrium latum* and other cestodes	Diphyllobo-thriasis (fish tapeworm) (16 million)	Infection from infected fish	Worldwide	Intestinal infection; Vit B12 deficiency
*Dipylidium caninum*	Dog tapeworm (rare)	Dogs and cats are main hosts; fleas intermediate hosts which can infect humans; bites	Worldwide	Intestinal infection
*Vampirolepis nana*	Dwarf tapeworm (36 million)	Rodents are main hosts; insects intermediate hosts; bites	Worldwide	Intestinal infection
(syn *. Hymenolepis)*				
*Taenia solium* ** **	Pork and beef tapeworm (80 million)	Infection from contaminated meat	Worldwide	Intestinal infection; cysts in various tissues (including brain)
*T. asiatica*				
*T. saginata*				
*Echinococcus granulosus*	Hydatidosis, echinococcosis (thousands)	Dogs, foxes;	Worldwide	Cyst (hydatid) formation in liver, lung or brain
*E. multilocularis*		Infection from faeces		

## 3. Antiparasitic Medicinal Plants and Their Secondary Metabolites

Parasites are eukaryotes and therefore share most molecular and biochemical properties with their eukaryotic hosts, making it often difficult to find antiparasitic drugs which are both effective and non-toxic for humans. This limitation always has to be kept in mind when discussing the numerous findings that some drug or extract from a medicinal plant is active against parasites *in vitro*. In order to be medicinally useful, such a drug must have bioavailability and should not intoxicate the patient. A first guidance is the determination of a selectivity index (SI) which compares the cytotoxicity of a drug against a parasite and a library of human cells.

### 3.1. Mode of Action of Antiparasitic Cytotoxic Drugs

A number of general cellular targets exists which can mediate cytotoxicity in human cells but also in parasites [8]. Major targets include: (1) DNA, RNA. (2) Proteins of the cytoskeleton. (3) Biomembranes.

#### 3.1.1. DNA, RNA

**Figure 1:**
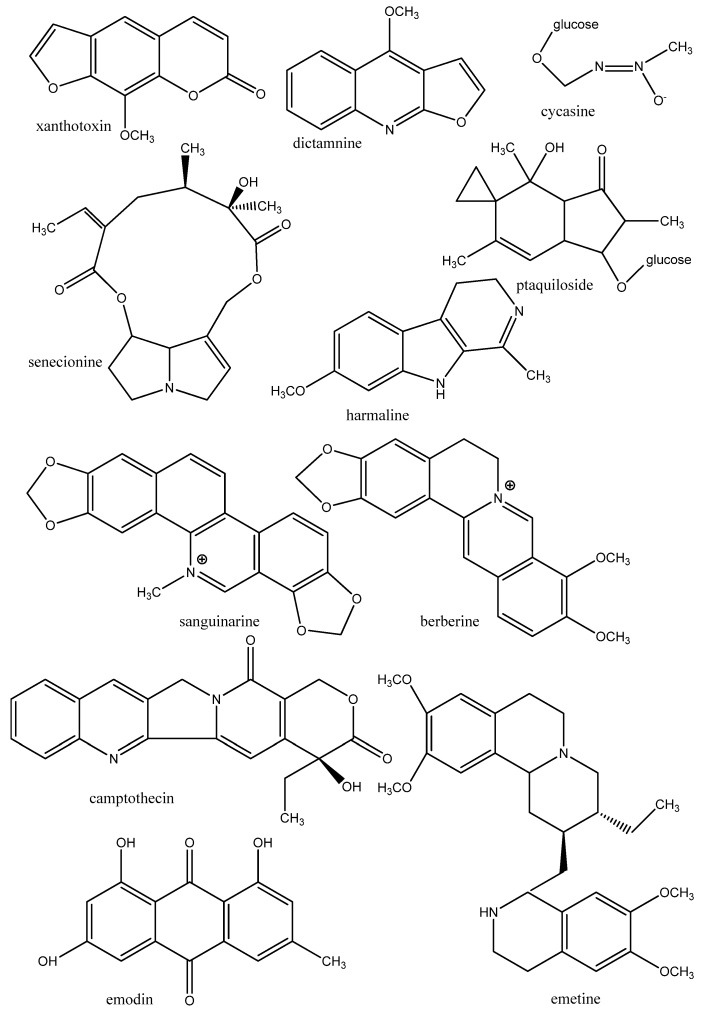
Examples of secondary metabolites which alkylate or intercalate DNA.

Compounds which damage DNA often have cytotoxic and antiparasitic properties. Typical DNA damage occurs when DNA alkylating compounds form covalent bonds with DNA bases. If these alkylations are not reversed by DNA repair enzymes, problems will occur after the next round of replication. Consequences are point mutations, and sometimes deletions and frame-shift mutations. If such mutations occur in important protein coding genes, they can lead to the death of a parasite. Typical DNA alkylating compounds which are found in medicinal plants include aristolochic acid (in *Aristolochia*), cycasin (in Cycadaceae), furanoquinoline alkaloids (in several species of Rutaceae), furanocoumarins (in many Apiaceae, Fabaceae), pyrrolizidine alkaloids (in Boraginaceae, Crotalarieae, Asteraceae), ptaquiloside (in *Pteridium aquilinum*) (Figure 1) and secondary metabolites (SM) with an epoxide as a functional group [9,10].

Another group of DNA damaging compounds intercalates DNA. These compounds are usually aromatic, planar and hydrophobic so that they can intercalate between the planar stacks of nucleotide pairs, especially GC-pairs. DNA intercalating compounds stabilize the DNA double helix and impair the replication process. Typical mutations which are caused by them are frame-shift mutations and deletions. These mutations usually lead to cell death [8]. Typical DNA intercalating compounds are common in the group of protoberberine and benzophenanthridine alkaloids, such as berberine and sanguinarine [10,11]. Many of the plants which produce such alkaloids (families Papaveraceae, Berberidaceae, Menispermaceae, Ranunculaceae) are known for their antiparasitic, antimicrobial, and antiviral properties. Intercalating alkaloids have also been detected among quinoline alkaloids (such as quinine), furanoquinoline alkaloids (Rutaceae), emetine (*Cephaelis acuminata*, Rubiaceae), beta-carboline alkaloids (e.g., in *Peganum harmala*, Nitrariaceae), anthraquinones (many Polygonaceae, Rhamnaceae), and furanocoumarins (many Apiaceae, Fabaceae) [9,10] (Figure 1).

Some of the intercalating compounds inhibit the enzyme DNA topoisomerase I or II which are essential for the replication process. If DNA topoisomerases are blocked, cells cannot divide. A typical topoisomerase inhibitor would be the indole alkaloid camptothecin (CPT) from *Camptotheca acuminata*, *Ophiorrhiza spp.*, *Notapodytes spp.*, *Ervatamia heyneana* and *Mostuea brunonis* [9,10,12] (Figure 1).

Cells treated with alkylating and intercalating drugs or topoisomerase inhibitors usually undergo programmed cell death by apoptosis [8] which can also occur in unicellular protozoa [13].

#### 3.1.2. Proteins of the Cytoskeleton and Enzymes

**Figure 2:**
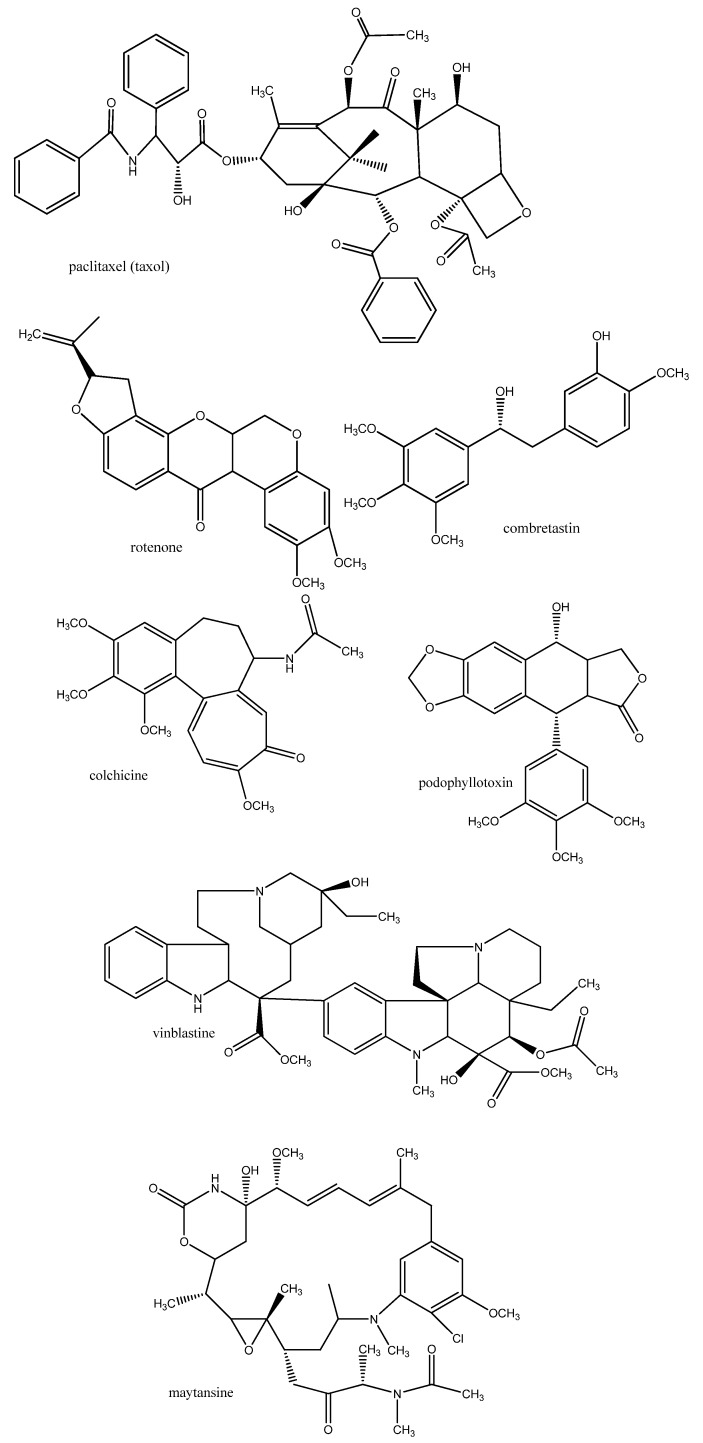
Examples of secondary metabolites which interfere with microtubule formation or disassembly.

Actin filaments and microtubules are the major proteins of the cytoskeleton of eukaryotic cells which are important for cell architecture. In addition, functional microtubules are required for the assembly of the mitotic spindle necessary for cell division. A number of natural products are known which have affinity for microtubules: some of them inhibit the polymerisation of tubulin into microtubules such as colchicine (from *Colchicum* spp., *Gloriosa superba*; Colchicaceae), vinblastine (*Catharanthus roseus*, Apocynaceae), podophyllotoxin (*Podophyllum* spp.; Berberidaceae; several *Linum* species; Linaceae), sanguinarine (*Sanguinaria canadensis*; *Macleaya* spp., *Bocconia* spp.; Papaveraceae) , maytansine (*Maytenus* spp.; Celastraceae), rotenone (in several genera of Fabaceae, such as *Derris* and *Lonchocarpus*), chalcones, combretastatin (in *Combretum caffrum*; Combretaceae) or inhibit the depolymerisation of microtubules (such as paclitaxel from *Taxus* spp.; Taxaceae) (Figure 2). Some of these natural products are presently used in the chemotherapy of cancer [8,12,14]. Often, they have antiparasitic properties.

The alkaloid emetine (from Rubiaceae) not only intercalates DNA but it is a very potent inhibitor of ribosomal protein biosynthesis. The pure alkaloid is still in use to treat amoebial infections [15]. Many parasites which live in the blood or intracellularly have closely adapted to their environment. These adaptations are often biochemically unique and offer vulnerable targets (enzymes, receptors) for specific antiparasitic drugs.

#### 3.1.3. Biomembranes

**Figure 3:**
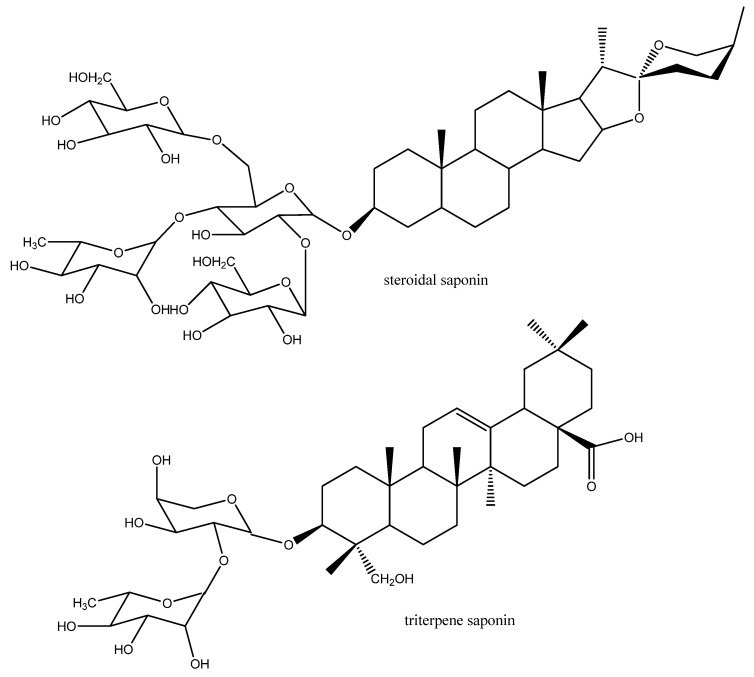
Example for steroidal and triterpene saponins (monodesmosides with one sugar chain).

All living cells are surrounded by a phospholipid bilayer, the cell membrane. It functions as a permeation barrier to prevent the escape of cellular metabolites but also controls an uncontrolled entry of polar or toxic exogenous compounds. If fluidity or permeability of the biomembrane are disturbed by natural products with detergent properties (as present in the large class of widely distributed triterpenoid and steroidal saponins, which occur in more than 30% of higher plants; Figure 3) a cytotoxic or antimicrobial effect can often be observed [9,16]. Small lipophilic secondary metabolites, such as terpenoids or phenylpropanoids as found in the essential oil of many plants (especially in Lamiaceae, Myrtaceae, Rubiaceae, Apiaceae, Asteraceae, Lauraceae, Rutaceae, Burseraceae, Verbenaceae, Pinaceae, Cupressaceae), can dissolve in biomembranes and disturb their fluidity and the function of membrane proteins [16]. Therefore, many of the lipophilic mono- and sesquiterpenes, phenylpropanoids and isothiocyanates (as present in Brassicaceae, Tropaeoleaceae) have a certain degree of antimicrobial and antiparasitic properties [9].

#### 3.1.4. Nervous System

Multicellular parasites have a nervous system with a number of important neurotransmitter/neuroreceptor systems, such as acetylcholine (ACh) and ACh-receptors (AChR). If the ACh-receptors, which control muscular activity, are inhibited or overstimulated, muscular paralysis can occur. This also happens if sodium and potassium channels are either blocked or stimulated, because ion signalling is essential in neuronal activity [17]. Muscular arrest can lead to direct death or in case of worms which are attached to the intestinal gut walls, they no longer can stick to the walls and are easily removed with the faeces after laxative treatment. The problem is to find a dose which affects the parasite but which is still not toxic for the patient. Cestodes and tapeworms which inhabit the intestine and not internal organs are easier to treat because any neurotoxic drug taken orally will affect them. The best compound would affect the worms but would not be absorbed by the intestinal cells. 

Alkaloids, a class with more than 21,000 compounds which occur in almost all plant families, are infamous for their neurotoxic properties [9,10,17]. Many of them are agonists or antagonists at neuroreceptors and/or ion channels [10,17]. They provide interesting candidates for anthelmintic drugs.

### 3.2. Anti-malaria Drugs

**Figure 4:**
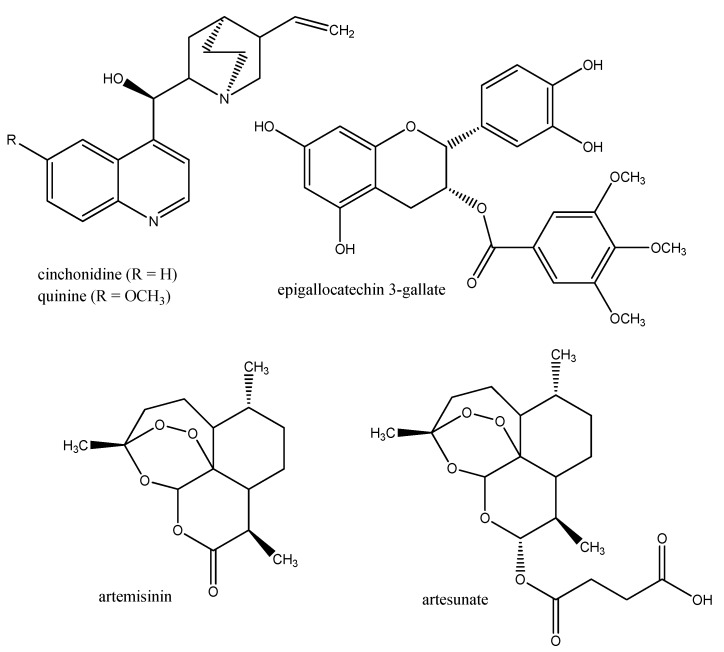
Examples of anti-malarial secondary metabolites. Artesunate is a semisynthetic derivative of artimisinine.

Human malaria is caused by unicellular sporozoa (Apicomplexa) of the genus *Plasmodium*, which are transmitted by various mosquito vectors (Table 1). More than 250 million people are infected and more than a million deaths (mostly among children) are been recorded annually. The first drugs to treat malaria came from *Cinchona officinalis* and related *Cinchona* species (Rubiaceae) which naturally occur in Central and South America. Extracts from Cinchona bark contain quinoline alkaloids, such as quinine, quinidine, cinchonine, and cinchonidine (administered as “Quinimax” in malaria therapy). It was especially the bitter-tasting quinine (Figure 4) which could be used to treat the blood stages of *Plasmodium* [1,18]. Quinine served as a lead structure for the synthesis of several antimalarial drugs such as chloroquine, mefloquine, pyrimethamine, proguanil, atovaquone (sold together with proguanil as “Malarone”), or primaquine. Quinine (alone or in combination with doxocycline, tetracycline or clindamycin) is still used today to treat acute cases of severe *P. falciparum* infections. Over the years *Plasmodium* (especially *P. falciparum* causing tropical malaria) has become resistant against many of the synthetic drugs. Among the mechanisms of drug resistance an enhanced expression of ABC transporters has been reported which can pump out any drug in an ATP dependent fashion that has entered the parasite [19].

A breakthrough for the development of antimalarial drugs was the identification of the sesquiterpene artemisinin from *Artemisia annua* (Asteraceae), which can even kill multidrug resistant strains of *P. falciparum* [18,20]. Several semisynthetic derivatives of artemisinin (e.g., the water soluble artesunate) have been developed which are in clinical practice today [21] (Figure 4). *A. annua* is an old medicinal plant, occurring from the Mediterranean, all over Asia to China, where this plant is used in TCM.

Among medicinal plants from all over the world, more than 1,200 have been used (at least at one stage) to treat malaria and fever. However, clinical trials are mostly missing [18]. Quite a large number of plants have been identified which produce natural products with significant antimalarial activity. An IC_50_ of less than 11 µM in *P. falciparum* usually indicates that a substance could be interesting for further drug development. Natural products with antimalarial activity have been identified among widely distributed phenolics (ellagic acid, epigallocatechin gallate, flavonoids, xanthones, coumarins, curcumin), naphthopyrones, quinones, widely distributed terpenoids (iridoids, sesquiterpenes, diterpenes, triterpenes), quassinoids, cucurbitacins (common in Cucurbitaceae), alkaloids (indolizidine, indole, isoquinoline), polyacetylenes [18,22,23,24,25,26,27,28,29,30,31,32,33,34].

Medicinal plants usually contain complex mixtures consisting of several classes of secondary metabolites. It has been postulated that the combinations found in these extracts exhibit synergistic interaction [16]. If epigallocatechin 3-gallate (EGCG), a typical polyphenol of green tea, is combined with the saponin digitonin, a synergistic reduction of motility and survival of *Plasmodium berghei* has been recorded [28]. Digitonin may facilitate the uptake of the polar EGCG. It might thus be a good strategy to look for synergistic combination partners in traditional medicines instead of focussing on single molecules [35,36].

### 3.3. Drugs against Trypanosomes (Trypanosoma, Leishmania)

#### 3.3.1. Trypanomiasis

Trypanosomes are widely distributed blood parasites of animals. Humans are infected by *T. brucei gambiense* and *T. b. rodesiense* causing sleeping sickness or Human African trypanosomiasis (HAT) (Table 1). *T. b. brucei* affects cattle and causes Nagana. *T. b. brucei* is often used in screening assays because this parasite does not infect humans and thus is of no health risk for the researcher. HAT and Nagana are transmitted by tsetse flies (*Glossina* spp.). HAT is restricted to tropical Africa where more than 60 million people are at risk of becoming infected. Vector control programs and improved public health control have reduced the number of infections from 0.5 million to approximately 50,000 to 70,000 patients, with 17,000 new infections per year [5,37,38].

Current chemotherapy of HAT is based on synthetic drugs developed more than 80 years ago, such as pentamidine and suramin. Whereas pentamidine binds to DNA, suramin is supposed to inhibit several glycolytic enzymes in trypanosomes. A derivative of pentamidine, pafuramidine (DB75) has clinically been tested recently. Later developed drugs include melarsoprol and eflornithine, the latter of which was registered in 1990. Eflornithine inhibits ornithine decarboxylase, which leads to a decrease of polyamine and of trypanothione, a unique antioxidant thiol compound of *Trypanosoma* and *Leishmania*. Eflornithine has recently been applied in combination with nifurtimox or melarsoprol. The number of chemotherapeutic drugs against HAT is very small and all of them exhibit severe side effects [5,37]. Furthermore, some trypanosomal strains have already become resistant to them, so that new drugs are urgently required.

A number of medicinal plants and secondary metabolites isolated from them have been screened for anti-trypanosomal activity [13,27,37,39,40,41,42,43,44,45,46]. Active natural products include several groups of alkaloids, phenolics, saponins, cardiac glycosides, other terpenoids, and polyacetylenes (common in Apiaceae, Asteraceae and Araliaceae). Although some natural products are active in the submicromolar range and show good selectivity, only few have been studied *in vivo* in an animal model. None of these results have been translated into clinical practice.

The mode of action of some drugs with anti-trypanosomal activities has been explored. A major target is glycolysis as blood forms can only gain energy by converting glucose to pyruvate or glycerol [38]. The enzymes responsible for the conversion of glucose to 3-phoshoglycerate and glycerol are found in a special compartment, the glycosome. If these enzymes are inhibited, trypanosomes will die. 

**Figure 5:**
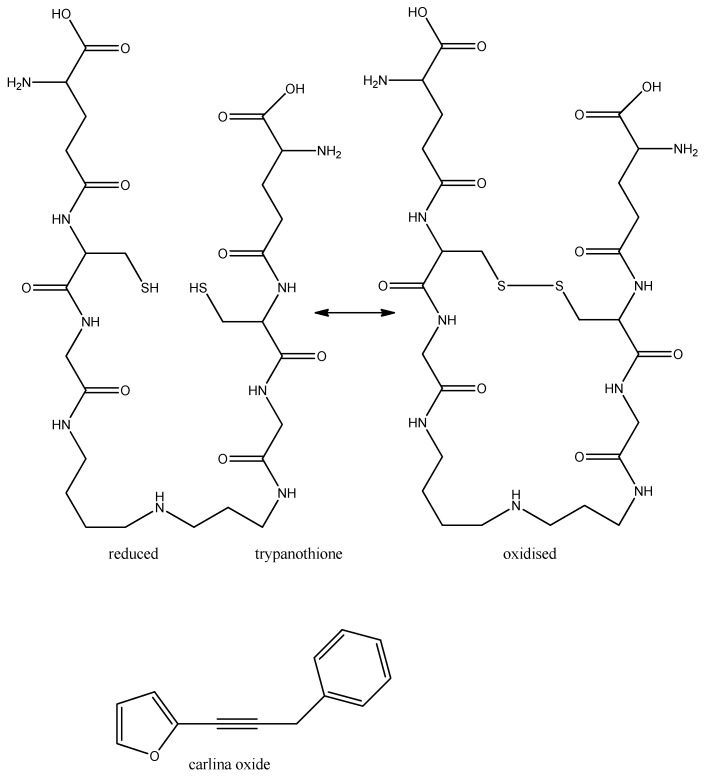
Reduced and oxidised form of trypanothione and carlina oxide which can block the SH-groups of trypanothione.

Trypanosomes lack catalase and glutathione peroxidases but have evolved a unique system with trypanothione to detoxify hydroperoxide. Trypanothione is built from two molecules of glutathione and one molecule of spermidine (Figure 5). Inactivation of the enzymes of trypanothione, spermidine or glutathione biosynthesis or of trypanothione directly will lead to death of the parasite [38]. The synthetic drug eflornithine (see above) inhibits ornithine decarboxylase which is important for spermidine biosynthesis. It is likely that secondary metabolites which can bind to the SH-group of trypanothione exhibit anti-trypanosomal activity. This has been shown for polyacetylenes which carry a reactive triple bond that can easily alkylate SH groups; e.g., the polyacetylene Carlina oxide from *Carlina acaulis* (Asteraceae) (Figure 5) and polyacetylenes from ginseng (*Panax ginseng*) have significant cytotoxic activity against *T. b. brucei* but are hardly toxic to human cells [47,48]. 

Trypanosomes have a single large mitochondrion, the kinetoplast, which contains several interlocked small (1 kb) and large (23 kb) circular mtDNA molecules (kDNA). Natural products which intercalate DNA target the sensitive kDNA. This has been shown in a comparative study with different groups of natural products [13,42]. Alkaloids which intercalate DNA such as berberine and sanguinarine exhibited IC_50_ values in the micromolar range [13]. Sanguinarine is also an inhibitor of microtubule formation [14].

Another target is DNA topoisomerase I; camptothecin, a known inhibitor of DNA Topo I also inhibits *T. brucei* with an IC_50_ of 1.5 µM [49]. The aporphine alkaloid dicentrine which is present in Papaveraceae and Lauraceae inhibits DNA Topo II and is active against trypanosomes [38,39]. Inhibitors of farnesyl transferase and tubulin polymerisation (e.g., vinblastine, sanguinarine) have substantial anti-trypanosomal activities [50].

#### 3.3.2. Chagas Disease

Infections with *Trypanosoma cruzi* cause Chagas disease on the American continent. These parasites are transmitted by triatomine bugs but also by blood transfusion, organ transplantation or contaminated food or drinks (Table 1). More than 28 million people are at risk, 15 million are infected and about 12,500 humans die annually from *T. cruzi* infections [5]. Patients with Chagas disease are treated with benznidazole, and nifurtimox, and both drugs exhibit severe side effects. Other drugs include protease x trans-sialase inhibitors and antagonists of ergosterol biosynthesis [5]. It is likely that many of the secondary metabolites, which have been tested against *T. brucei* [13,42] also affect *T. cruzi* [5].

#### 3.3.3. Leishmaniasis

Leishmaniasis is caused by protozoan parasites of the genus *Leishmania* (Table 1) which invade macrophages of host organisms. A distinction is made between cutaneous, mucocutaneous, visceral and diffuse leishmaniasis, of which visceral leishmaniasis is a fatal disease causing approximately 60,000 death per year [51]. Leishmaniasis and HIV infections often co-occur and these patients usually have a poor prognosis. Patients are treated with the synthetic drugs stibogluconate, meglumine and pentamidine (developed 70 years ago), which have severe side effects and fail to work in North Bihar (India) [5,52]. Also the macrolide antibiotic amphotericin B has been employed, which can also be toxic for patients. New developments include the anticancer drug miltefosine, the aminoglycoside antibiotic paronomycin, and the 8-aminoquinoline sitamaquine [51]. Among natural products, berberine (which occurs in many TCM plants) had promising anti-leishmanial activities [5,52].

It is relatively easy to cultivate the promastigote form (which lives in the insect) axenically and to determine the cytotoxic effects of extracts or isolated compounds either by counting the parasites, or MTT or Alamar Blue photometric assays. More complicated is the analysis of the less susceptible amastigote forms in macrophages but these data are more realistic than those obtained from promastigote parasites. Transgenic amastigote forms which express reporter genes such as GFP have facilitated the screening [51,53].

A number of natural products have already been screened. Among natural products from marine sources cyclic peptides, various flavonoids, chalcones, lignans, coumarins, iridoids, monoterpenes, saponins, toxoids, curcumin, quinoline alkaloids, and polyketides exhibit interesting anti-leishmanial activities [27,51,52]. Active flavanones and flavonoids were reported from *Baccharis retusa* (Asteraceae) and *Kalanchoe pinnata* (Crassulaceae) [51].

### 3.4. Drugs against Trichomonas Vaginalis

Trichonomiasis is caused by *Trichomonas vaginalis* and constitutes a common sexually-transmitted protozoan infection (Table 1). It mostly affects women and to minor degree men; about 170 million people are infected worldwide. It is often associated with HIV and cervical cancer [54]. Standard chemotherapy includes 5-nitroimidazoles, such as metronidazole and tinidazole. Because some of the *Trichomonas* strains have become resistant to the standard drugs, new chemicals are needed. Natural products have been analysed as an alternative and several alkaloids (e.g., berberine), dibenzofurans, anthraquinones, polyacetylenes, saponins, and diterpenes [54].

### 3.5. Drugs against Worms (Nematoda, Cestoda, Trematoda)

As illustrated in Table 1, a large diversity of worms belonging to different classes of invertebrates (Nematoda, Cestoda, Trematoda) can parasitize humans. Especially lymphatic filariasis, onchocerciasis, loaisis, schistosomiasis and other helminth-mediated diseases affect several million humans. It has been estimated that probably two billion humans (28% of mankind) are infected by at least one species of helminth parasite at some stage of life [55,56]. The prevalence can be much higher in subtropical and tropical developing countries with poor hygiene and limited access to expensive medications.

A number of *in vitro* and *in vivo* assays for anti-filarial and anti-helminthic screening have been developed, in which motility and viability are evaluated [56]. But because of the multitude of parasites involved adequate experimental systems are not available for every relevant parasite or relevant life stage.

Lymphatic filariasis is currently being treated with diethylcarbamazine (DEC) and ivermectin or in combination with albendazole [55]. New drugs are urgently required in order to treat drug resistant filariasis. A number of plants from traditional medicine, including plants from Ayurvedic and Chinese medicine have already been screened and a number of promising drugs or natural products could be identified [56,57]. *Streblus asper* (Moraceae) has proven anti-filarial activity both *in vitro* and *in vivo* and has been tested clinically in India [58]. The cardiac glycosides asperoside and strebloside appear to be the active components [59]. 

Onchocerciasis is treated with ivermectin in combination with albendazole whereas none is known for dracunculiasis [55]. The number of plants tested against onchocerciasis and dracunculiasis is much smaller than for filariasis, but a few African plants have been recorded [56,60]. In most studies only extracts from medicinal plants were screened and the profiles of secondary metabolites present were not established with modern phytochemical methods. 

**Figure 6:**
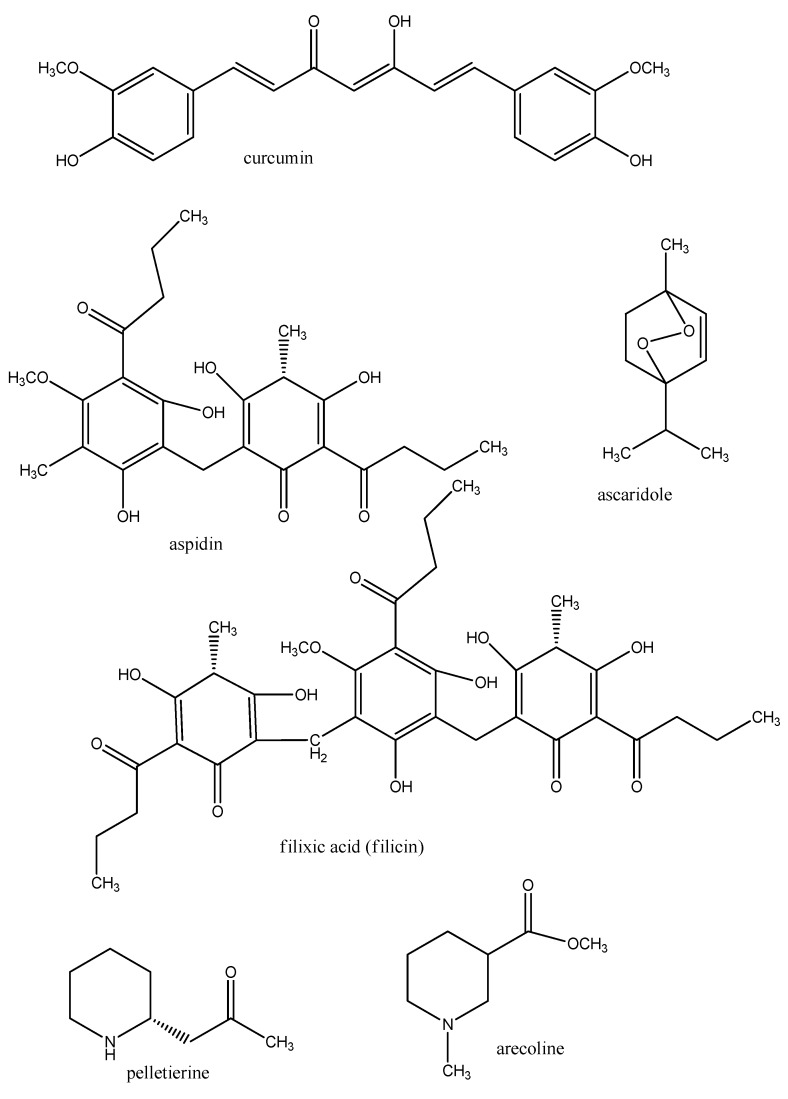
Secondary metabolites with anthelmintic properties.

Schistosomiasis is treated with praziquantel alone or in combination with albendazole or ivermectin. Also oxamniquine, and antimalarial drugs, such as quinoline alkaloids and artemisinin and its derivatives have been employed [55]. Anthraquinones in *Rheum palmatum* and *Rumex dentatus* (Polygonaceae), phorbolesters from *Jatropha curcas* (Euphorbiaceae), and saponins in general exhibited molluscicidal activity against schistosomias vector snails *Oncomelania*, *Biomphalaria* and *Bulinus* [61]. Curcumin (Figure 6) and derivatives from *Curcuma* plants (Zingiberaceae) are parasiticidal in *Schistosoma* [27].

Parasitic intestinal helminths have been treated with vermicides and vermifuges. A synthetic drug is mebendazol that has been developed 40 years ago. It inhibits microtubules in the intestinal cells of worms, which leads to their degeneration and malnutrition. Mebendazol is only partly absorbed from human intestines and eliminated by first pass effects. Another synthetic anthelmintic is ivermectin. Important plants for this indication comprise *Chenopodium ambrosioides* (Amaranthaceae) and ascaridole which has been isolated from this plant [62] (Figure 6). Ascaridole is effective against hookworm infection, but mutagenic and poisonous [9,10].

Another traditional herb is the fern *Dryopteris filix-mas* (Dryopteridaceae) which contains vermicidal phloroglucinols, such as aspidin, deaspidin, and filixic acid (syn. filicin) (Figure 6). They are active against intestinal cestodes and probably paralyze the worm’s muscles [1]. Also this drug has considerable side effects for humans but filixic acid is used as an anthelmintic in veterinary praxis. Other paralyzing agents are the anthelmintic alkaloids pelletierine from *Punica granatum* (Lythraceae) and arecoline from *Areca catechu* (Arecaceae), which target acetylcholine receptors [9,10] (Figure 6).

Other anthelmintic plants include *Artemisia maritima* (with santonin), *Artemisia abrotanum* (Asteraceae) *Zanthoxylum liebmannianum* (Rutaceae), *Thymus vulgaris* (Lamiaceae), *Millettia thonningii*, *Albizzia anthelmintica*, *Butea frondosa* (Fabaceae), *Embelia schimperi* (Myrsinaceae), *Teloxys graveolens* (Amaranthaceae) and several others [56].

## 4. Conclusions

In this review the relevant parasitic infections of humans and standard medications are tabulated. Several of the parasites have become resistant to chemotherapy, so alternatives are urgently required. Since vaccination has failed in most instances, the search for small molecules is still an option. For malaria and trypanosomiasis quite a number of medicinal plants and isolated natural products have already been tested, but for most of the other parasitic diseases such information is largely missing. Most of the antiparasitic properties of extracts and isolated natural products have been tested *in vitro* only. Translation of the *in vitro* research results into *in vivo* trials is urgently required. Furthermore, even if animal experiments were successful, we would need clinical trials of the new compounds alone or in combination with established parasiticidal drugs to prove their efficacy and safety. These developments are costly and it is presently difficult to attract the pharmaceutical industries into these fields for various reasons.

TCM and other traditional medicine systems employ several thousand of medicinal plants; some of which have known antiparasitic properties. They offer a unique opportunity to identify natural products which could be potentially used to treat parasitic infections. The success story of artemisinin from *Artemisia annua* [2,20,63] can probably be repeated.
